# A *DELLA* gene, *RhGAI1*, is a direct target of EIN3 and mediates ethylene-regulated rose petal cell expansion via repressing the expression of *RhCesA2*

**DOI:** 10.1093/jxb/ert296

**Published:** 2013-09-07

**Authors:** Jing Luo, Nan Ma, Haixia Pei, Jiwei Chen, Jing Li, Junping Gao

**Affiliations:** Department of Ornamental Horticulture, China Agricultural University, Beijing 100193, China

**Keywords:** Cell expansion, ethylene, petal, *RhGAI1*, rose, transcriptional regulation.

## Abstract

Ethylene plays an important role in organ growth. In *Arabidopsis*, ethylene can inhibit root elongation by stabilizing DELLA proteins. In previous work, it was found that ethylene suppressed cell expansion in rose petals, and five unisequences of *DELLA* genes are induced by ethylene. However, the mechanism of transcriptional regulation of *DELLA* genes by ethylene is still not clear. The results showed that the expression of *RhGAI1* was induced in both ethylene-treated and *ETR* gene-silenced rose petals, and the promoter activity of *RhGAI1* was strongly induced by RhEIN3-3 in *Arabidopsis* protoplasts. What is more, RhEIN3-3 could bind to the promoter of *RhGAI1* directly in an electrophoretic mobility shift assay (EMSA). Cell expansion was suppressed in *RhGAI1-Δ17*-overexpressed *Arabidopsis* petals and promoted in *RhGAI1*-silenced rose petals. Moreover, in *RhGAI1*-silenced petals, the expression of nine cell expansion-related genes was clearly changed, and *RhGAI1* can bind to the promoter of *RhCesA2* in an EMSA. These results suggested that *RhGAI1* was regulated by ethylene at the transcriptional level, and *RhGAI1* was a direct target of RhEIN3-3. Also, *RhGAI1* was shown to be involved in cell expansion partially through regulating the expression of cell expansion-related genes. Furthermore, *RhCesA2* was a direct target of *RhGAI1*. This work uncovers the transcriptional regulation of *RhGAI1* by ethylene and provides a better understanding of how ethylene regulates petal expansion in roses.

## Introduction

Rose is one of the most important commercial flowers worldwide. In China, 4.1 billion stems of cut roses were sold in 2010, accounting for 38% of total sales of cut flowers (data from the Ministry of Agriculture of China). Cut flowers of roses are usually harvested at the bud stage, which is helpful in reducing physical damage to the petals in post-harvest handling, such as packing and transportation. It is reported that post-harvest loss of cut roses is mainly caused by ethylene, which can result in abnormal flower opening (Ma *et al.*, 2006; Xue *et al.*, 2008). Therefore, investigation of the underlying mechanism of how ethylene regulates rose flower opening has been an important issue in post-harvest biology of ornamental plants worldwide.

Ethylene plays an important role in multiple aspects of plant growth and development, including seed germination, seedling growth, flower opening, fruit ripening, and senescence (Zhao and Guo, 2011). In regulation of plant organ growth, ethylene can function in opposite ways (Pierik *et al.*, 2006). In most cases, ethylene inhibits organ growth, such as root elongation (Swarup *et al.*, 2007), hypocotyl elongation, and cotyledon expansion (Hall and Bleecker, 2003) in *Arabidopsis*, and internode elongation in rice (Qi *et al.*, 2011). In some other cases, ethylene stimulates organ growth, such as cell elongation in cotton fibre (Shi *et al.*, 2006) and internode elongation in rice in response to deep water (Hattori *et al.*, 2009). However, the mechanisms underlying petal growth regulation by ethylene are not well understood.

Transcription factors are widely involved in phytohormone signalling, and regulate plant growth and development. In terms of ethylene-regulated organ growth, BOLITA, an AP2/ERF1 transcription factor, represses both cell proliferation and cell expansion in *Arabidopsis* (Marsch-Martinez *et al.*, 2006).

DELLA proteins, a subfamily of GRAS transcription factors, also play important roles in plant growth and development. In *Arabidopsis*, there are five DELLA proteins: GA INSENSITIVE (GAI), REPRESSOR OF *ga1-3* (RGA), and three RGA-LIKE proteins (RGL1, RGL2, and RGL3) (Harberd *et al.*, 2009). Among them, GAI and RGA have high redundancy in their function, and they both repress gibberellic acid (GA)-regulated organ growth (Wang *et al.*, 2009).

Plant organ growth is coordinated in two processes, cell proliferation and cell expansion (Geitmann and Ortega, 2009; Krizek, 2009). In leaf growth of *Arabidopsis*, DELLA proteins not only inhibit the cell division rate during the proliferation phase, but also repress the cell expansion rate during the expansion phase (Achard *et al.*, 2009; Gonzalez *et al.*, 2012). To date, however, whether the DELLA proteins are involved in petal expansion remains unclear.

Petal growth depends on cell division during early development stages, but is regulated by cell expansion during late flower development (Varaud *et al.*, 2011). In *Arabidopsis*, it undergoes the transition from cell division to cell expansion during stage 9–11 of flower opening. After stage 12, flower opening is mainly attributed to cell expansion (Irish, 2008). In cut roses, flowers are harvested commercially at stage 2—the bud-opened stage—and the petal expansion depends on cell expansion (Yamada *et al.*, 2009).

Cell expansion is accompanied by modification of the cell wall, changes of turgor pressure, and remodelling of the cytoskeleton (Winship *et al.*, 2010). It is reported that ethylene influences cell expansion partly through regulating the expression of cell expansion-related genes. Ethylene induces the expression of *XTH* and *EXPANSIN7*, which results in cell wall loosening and root hair initiation in *Arabidopsis* (Sánchez-Rodríguez *et al.*, 2010). Ethylene can repress the expression of an aquaporin gene, *RhPIP2;1*; thus, it partly contributes to the inhibition of water absorption and cell expansion in rose petals (Ma *et al.*, 2008). However, to date, how these genes are regulated by ethylene and whether *DELLA* genes mediate this process remains unclear.

Here, an ethylene-responsive *DELLA* gene, *RhGAI1*, was isolated through microarray analysis. The results indicated that *RhGAI1* was regulated by ethylene at the transcriptional level, and *RhGAI1* was a direct target of RhEIN3-3. In addition, *RhGAI1* was found to be involved in cell expansion partly through regulating the expression of several cell expansion-related genes, and *RhCesA2* was a direct target of RhGAI1.

## Materials and methods

### Plant materials and growth conditions

The flowers of cut roses (*Rosa hybrida*) cv. Samantha were harvested at stage 2 from the greenhouse, and transported to the laboratory within 1h. The flower stems were cut to 25cm length in distilled water and then placed in vases with distilled water. The ethylene and 1-methylcyclopropene (1-MCP) treatment were according to Ma *et al.* (2008).

Seeds of *Arabidopsis thaliana* were surface sterilized and sown on Murashige and Skoog (MS) medium. After vernalization, the seeds were transferred to a growth chamber for 7 d. The 7-day-old seedlings were transplanted into growth mixture (vermiculite/nutritive soil=1:1). For protoplast extraction, the wild-type (Col-0) seedlings were grown under conditions with a photoperiod of 12h light/12h dark at 23 °C, low light intensity (50–75 mE m^–2^ s^–1^), and a relative humidity of 40–60%.

### Plasmid construction and plant transformation

For silencing of *RhGAI1*, a 321bp *RhGAI1* 3′-untranslated region sequence was amplified from the cDNA sample of rose flowers, and inserted into the multiple cloning site (MCS) of *pTRV2* to construct the *pTRV2-RhGAI1* vector. For silencing of *RhETR* genes, a fragment of 852bp, possessing the conserved domain of *RhETR1*, *RhETR3*, and *RhETR5*, was amplified from rose cDNA, and inserted into *pTRV2* to construct the *pTRV2-RhETR*s vector. The silencing of *RhGAI1* and *RhETR* genes in petals by virus-induced gene silencing (VIGS) was performed according to the procedures described by Dai *et al.* (2012).

The *pSuper::GFP-RhGAI1-Δ17* vector was constructed by overlapping PCR. In *RhGAI1-Δ17*, the 51bp fragment encoding the DELLA domain was deleted, mimicking the mutation of *gai*. The recombined *pSuper::GFP-RhGAI1-Δ17* vector was transformed into *Agrobacterium* strain GV3101 and then introduced into *Arabidopsis* plants using the ﬂoral dip method (Clough and Bent, 1998).

To obtain the *pUC-35S mini::GUS* vector, the sequence harbouring the 35S mini promoter and β-glucuronidase (GUS) gene was excised from the pBI-89 vector (Liu *et al.*, 2003), and subcloned into the pUC19 vector. The promoter regions of *RhCesA2* and *RhPIP2;1* were inserted into the *pUC-35S mini::GUS* vector to replace the *35S mini* promoter, and the *pUC*-pro*RhCesA2::GUS* and *pUC*-pro*RhPIP2;1::GUS* reporter vectors were generated.

For prokaryotic expression of RhEIN3-3 and RhGAI1, the N-terminus (amino acids 141–356) containing the binding domain of RhEIN3-3 (Shi *et al.*, 2012) and the C-terminus (amino acids 238–618) containing the binding domain of RhGAI1 (Hirano *et al.*, 2010) were inserted into the pGEX-2T vector.

### RNA extraction and quantitative real-time PCR (qRT-PCR) analysis

The total RNA of rose petals was extracted using the hot borate method, according to Ma *et al.* (2006). The primer sequences are listed in Supplementary Table S1 available at *JXB* online. *RhACT5* was used as the internal control. The qRT-PCR analysis was done with the KAPA SYBR^®^ FAST qPCR Kits (KAPA BIOSYSTEMS, USA) on an ABI7500 machine (Applied Biosystems by Life Technologies, USA).

### Microscopic examination and cell counting

Observation of abaxial subepidermis (AbsE) cells was performed according to Ma *et al.* (2008). The number of AbsE cells in a 388×388 μm area was counted and statistical analysis was performed with SPSS software.

For *Arabidopsis*, petals of flowers at stage 14 (Smyth *et al.*, 1990) were fixed by FAA and used to count cell numbers. The number of AbsE cells in a 261×261 μm area was counted, and statistical analysis was performed with SPSS software.

### Sequence analysis

Alignment of the deduced amino acid sequence was performed using ClustalX (default values were used) and DNAMAN (default values were used), and phylogenetic analysis was performed using ClustalX and MEGA. The phylogenetic trees were computed using the Neighbor–Joining algorithm with 10 000 bootstrap replicates.

### Subcellular localization

The *pSuper::GFP-RhGAI1* and *pSuper::GFP* vectors were bombarded into onion bulb scale epidermal cells. After incubation in the dark at 23 °C for 24h, the onion epidermis was stained with 2.5 μg ml^–1^ 4′,6-diamidino-2-phenylindole (DAPI) for 30min. Fluorescence signals were detected using a Nikon T1 confocal laser-scanning microscope (Nikon, Japan). The excitation wavelengths for green fluorescent protein (GFP) and DAPI were 488nm and 408nm, respectively, and the emission filter wavelengths were 505–530nm for GFP and 420–480nm for DAPI.^4^

### Transient expression in *Arabidopsis* mesophyll protoplasts

The *Arabidopsis* mesophyll protoplasts were prepared according to Yoo *et al.* (2007). The plasmids were extracted with MACHEREY-NAGEL kits; 10 μg of effector plasmid and 10 μg of reporter plasmid were introduced into ~2×10^4^ protoplasts with polyethylene glycol (PEG). The GUS activity was determined according to Li *et al.* (2009).

### Electrophoretic mobility shift assay (EMSA)

The EMSA was performed according to Wang *et al.* (2011). Recombinant pGEX-RhEIN3-3 and pGEX-RhGAI1 proteins were produced in *Escherichia coli* strain BL21. The *E. coli* cells were lysed by sonication, and purified with glutathione–Sepharose 4B beads (GE Healthcare).

The *proRhGAI1* probe 5′-CGTTTTATACTAATTCAAA GGTATTAA-3′, the *proRhCesA2* probe 5′-GCAGAGGGCTAA TTTAGTCGGTGGAGAT-3′, and their complementary probes were labelled with biotin. A 1 μg aliquot of recombinant protein and 2nM biotin-labelled probe were used for the binding reaction for each sample. The LightShift chemiluminescent EMSA kit (Pierce, IL, USA) was used for EMSA.

## Results

### Isolation of *RhGAI1*

In order to investigate the molecular mechanism underlying the ethylene-regulated flower opening in rose, a microarray database (http://bioinfo.bti.cornell.edu/rose) was built. Five out of eight unisequences, annotated as DELLA proteins, were found to be significantly up-regulated by ethylene at early times (Supplementary Table S2 at *JXB* online).

**Fig. 1.**
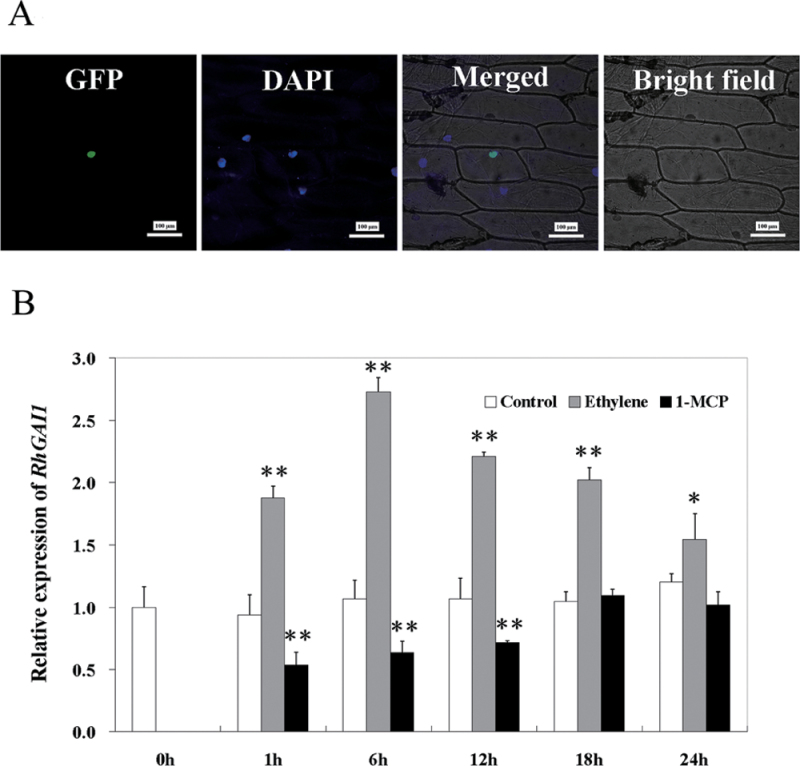
(A) Subcellular localization of *RhGAI1* in onion epidermal cells. Scale bar=100 μm. (B) qRT-PCR analysis of *RhGAI1* in ethylene- and 1-MCP-treated rose petals. The results show the mean and SD (error bars) for data from three biological replicates. *RhACT5* was used as the internal control. The relative expression of *RhGAI1* in control petals was set to 1. Asterisks indicate a significant difference between ethylene/1-MCP and control. Student’s *t*-test, *P < 0.05; **P < 0.01.

By using rapid amplification of cDNA ends (RACE), these five unisequences were found to be different parts of the same gene, which encoded a DELLA protein with 618 amino acid residues. Phylogenetic analysis showed that this DELLA protein had high homology with MdGAI1, MhGAI1, and AtGAI. Therefore, the gene was named *RhGAI1* (GenBank accession no. AGK07287) (Supplementary Figs S1, S2 at *JXB* online). The *pSuper::GFP-RhGAI1* plasmid was then transferred into onion (*Allium cepa*) epidermal cells by particle bombardment. The result indicated that RhGAI1 was located in the nucleus (Fig. 1A).

### *RhGAI1* is transcriptionally regulated by ethylene

The expression of *RhGAI1* in petals was determined by qRT-PCR. The results showed that, compared with untreated control, ethylene significantly enhanced the expression of *RhGAI1* by 1.9-fold at 1h and by 2.75-fold at 6h. 1-MCP, an inhibitor of ethylene action, significantly reduced the expression of *RhGAI1* within 12h of treatment (Fig. 1B).

**Fig. 2.**
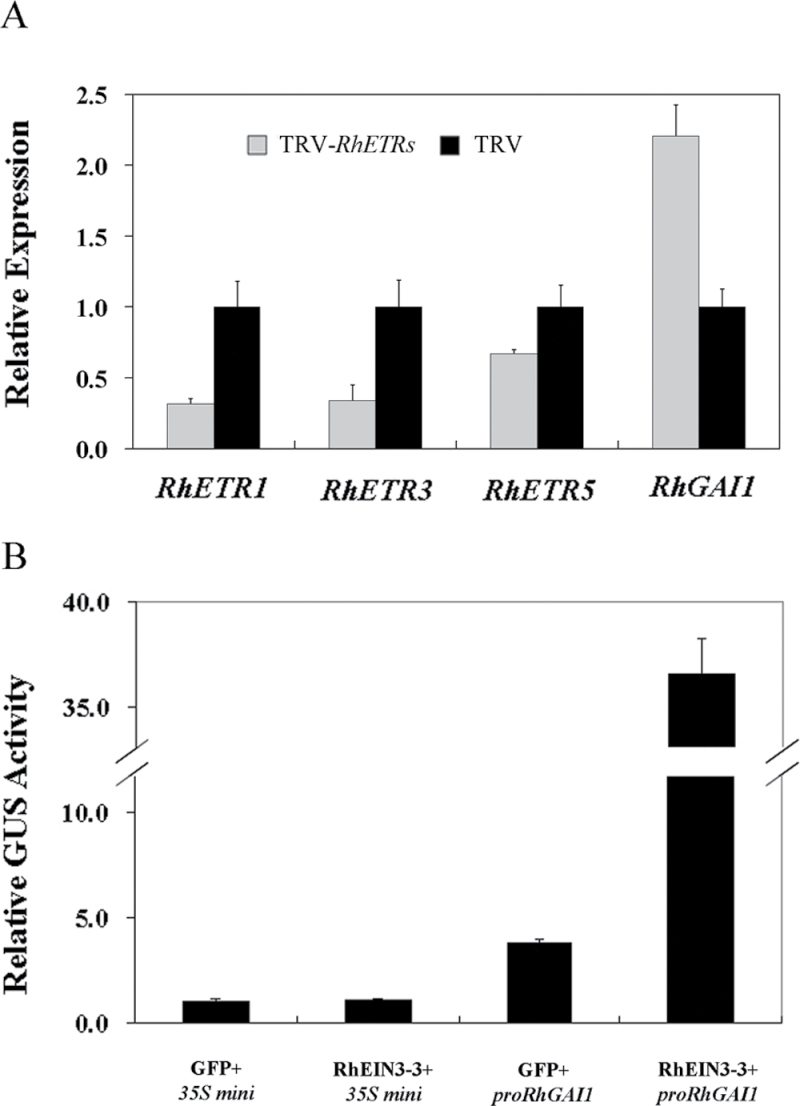
Transcriptional regulation of *RhGAI1* by ethylene. (A) qRT-PCR analysis of *RhGAI1* in *RhETR* gene-silenced rose petals. The results show the mean and SD from three biological replicates. The relative expression of *RhGAI1* and *RhETR* genes in TRV samples was defined as 1. (B) Regulation of the *RhGAI1* promoter activity by RhEIN3-3 in *Arabidopsis* mesophyll protoplasts. The *35S mini* promoter was used as the control, and its relative GUS activity was defined as 1.

Ethylene receptors (ETRs) function as negative regulators in ethylene signalling, and five members have been identified in rose flowers (Tan *et al.*, 2006). To check whether *RhGAI1* acted downstream of ethylene signalling, the *RhETR* family was silenced in rose petals through the VIGS approach. The expression of *RhGAI1* was clearly increased in *RhETR* gene-silenced petals when compared with the *Tobacco rattle virus* (TRV) control (Fig. 2A). The results above suggested that *RhGAI1* was regulated by ethylene at a transcriptional level.

**Fig. 3.**
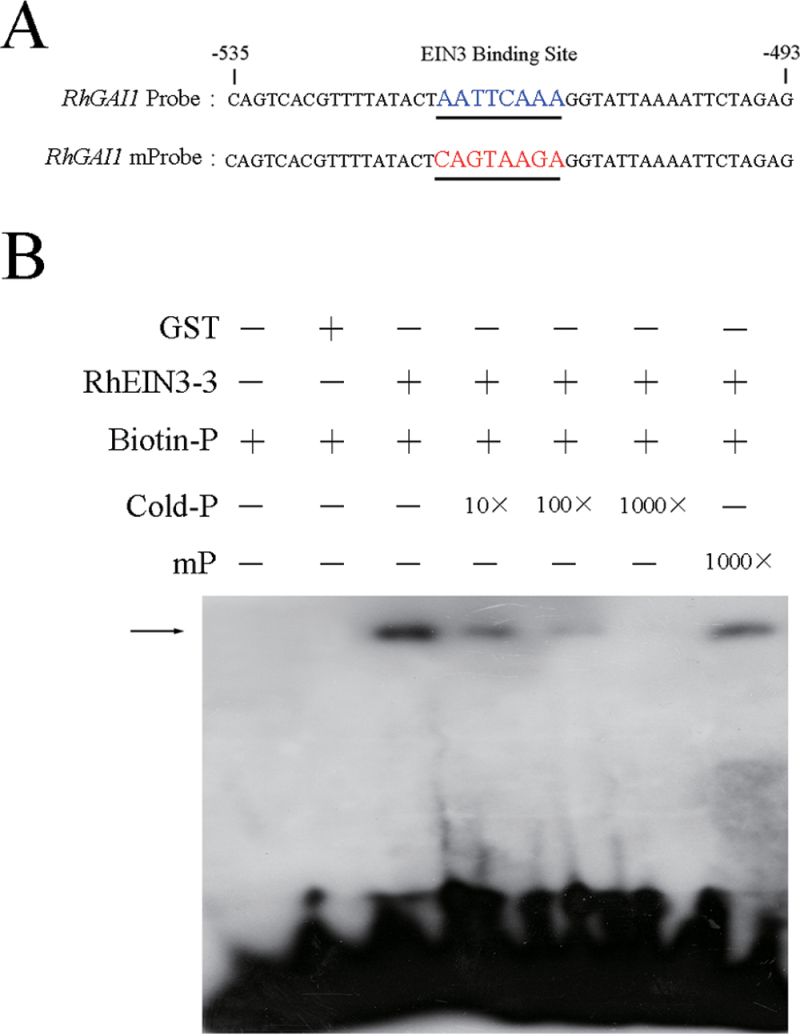
Binding test of RhEIN3-3 and the probe of *proRhGAI1 in vitro*. (A) The *RhGAI1* probe was labelled with biotin. (B) EMSA analysis showing binding of RhEIN3-3 to the *RhGAI1* promoter. The black arrow indicates the binding of RhEIN3-3 and the biotin-labelled *RhGAI1* probe.

Through sequence analysis of the 1507bp promoter sequence of *RhGAI1*, an EIN3 binding site (EBS) ‘AATTCAAA’ was found in the region from –518bp to –511bp of *proRhGAI1* (Fig. 3A).

EIN3 is known as the primary transcription factor in ethylene signalling (Zhao and Guo, 2011). Based on previous transcriptome sequencing, seven unisequences of *EIN3* genes were obtained (Supplementary Table S3 at *JXB* online). The unisequence *RU03799*, which had higher homology with *EIN3* in other plants, was cloned by RACE. The full length of *RU03799* was 1857bp, encoding a deduced protein with 618 amino acids. Phylogenetic analysis showed that the deduced protein had higher homology with CsEIN3 and RcEIN3, and the gene was named *RhEIN3-3* (GenBank accession no. AGK07288) (Supplementary Fig. S3), since two rose *EIN3* genes, *RhEIN3-1* and *RhEIN3-2*, have been deposited in GenBank.

To test whether *RhGAI1* was regulated by RhEIN3-3, a transactivation test was performed in *Arabidopsis* mesophyll protoplasts. The promoter activity of *RhGAI1* was strongly induced by RhEIN3-3, compared with the vector control (Fig. 2B). These results indicated that *RhGAI1* might be a downstream gene of *EIN3-3*.

Further, an EMSA was carried out to test the direct binding of *proRhGAI1* and RhEIN3-3. The biotin-labelled probe was designed according to the EBS element in the promoter of *RhGAI1* (Fig. 3A). The EMSA result showed that RhEIN3-3 could bind to the biotin-labelled probe of *proRhGAI1*. Also, this binding was attenuated gradually by increasing the concentration of unlabelled probe, whereas it could not be competed by the mutated probe (Fig. 3B). This result indicated that *RhGAI1* was a direct target of RhEIN3-3.

### *RhGAI1* is involved in cell expansion in both *Arabidopsis* and rose petals

**Fig. 4.**
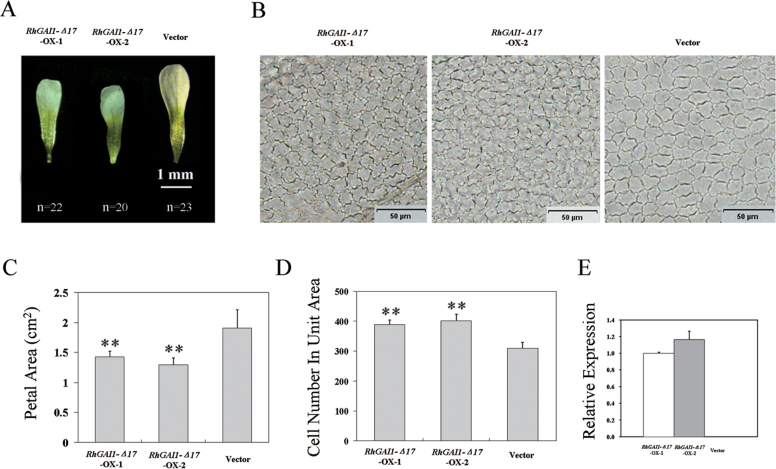
Overexpression of *RhGAI1-Δ17* in *Arabidopsis*. (A) Petal and (B) AbsE cell phenotype of *RhGAI1-Δ17*-OX transgenic lines and the vector. Determination of (C) the petal area and (D) cell numbers in transgenic lines and the vector. Each plot represents the mean ±SD, *n*=22, 20, and 23, respectively. Student’s *t*-test, **P < 0.01. (E) qRT-PCR analysis of *RhGAI1* in transgenic lines and the vector. The results show the mean ±SD from three biological replicates.

To understand whether *RhGAI1* functions in cell expansion, *RhGAI1* was overexpressed in *Arabidopsis*, and the function of *RhGAI1* in expansion of petals was investigated. As DELLA proteins are easily degraded by GA, gain-of-function mutagenesis of *RhGAI1* was carried out, and *RhGAI1-Δ17* was obtained, in which 17 degradation-related amino acids in the DELLA domain were deleted (Dill *et al.*, 2001). Compared with the vector control, the expansion of petals and AbsE cells was markedly repressed in *RhGAI1-Δ17*-overexpressing (OX) lines (Fig. 4A, B, E). The petal area was 1.90±0.30mm^2^ in the vector control, and decreased to 1.43±0.09mm^2^ and 1.30±0.11mm^2^ in *RhGAI1-Δ17*-OX-1 and OX-2 petals, respectively (Fig. 4C). In a 261×261 μm area of petals, the average cell number was 308.3±18.7 in the vector control, and 388.6±15.7 and 402.7±20.5 in OX-1 and OX-2 petals, respectively (Fig. 4D). These results indicated that *RhGAI1* was involved in inhibition of cell expansion in petals of *Arabidopsis*.

**Fig. 5.**
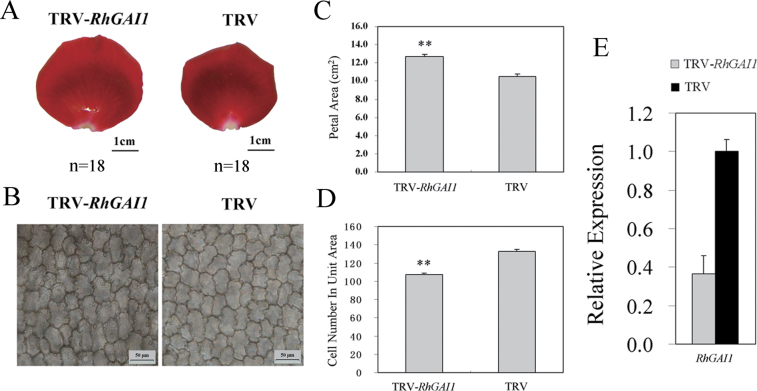
Silencing of *RhGAI1* in rose petals by VIGS. (A) Petal and (B) AbsE cell phenotype of *RhGAI1*-silenced samples and the TRV control. Determination of (C) the petal area and (D) cell numbers in *RhGAI1*-silenced samples and the TRV control. Each plot represents the mean ±SD, *n*=18 for both. Student’s *t*-test, **P < 0.01. (E) qRT-PCR analysis of *RhGAI1* in silenced petals and the TRV control. The results show the mean ±SD from three biological replicates.

*RhGAI1* was also silenced in rose petals by VIGS. The expansion of petals was substantially promoted in *RhGAI1*-silenced samples when compared with the TRV control (Fig. 5A, E). The average size in control petals was 10.52±1.02cm^2^, and 12.72±0.80cm^2^ in *RhGAI1*-silenced petals (Fig. 5C). In a 388×388 μm area of petals, the average cell number was 1032.3±12.23 in control petals, and 107.5±4.8 in *RhGAI1*-silenced petals (Fig. 5B, D). These results indicated that *RhGAI1* was involved in repression of cell expansion in rose petals.

### *RhGAI1* regulates several cell expansion-related genes

**Fig. 6.**
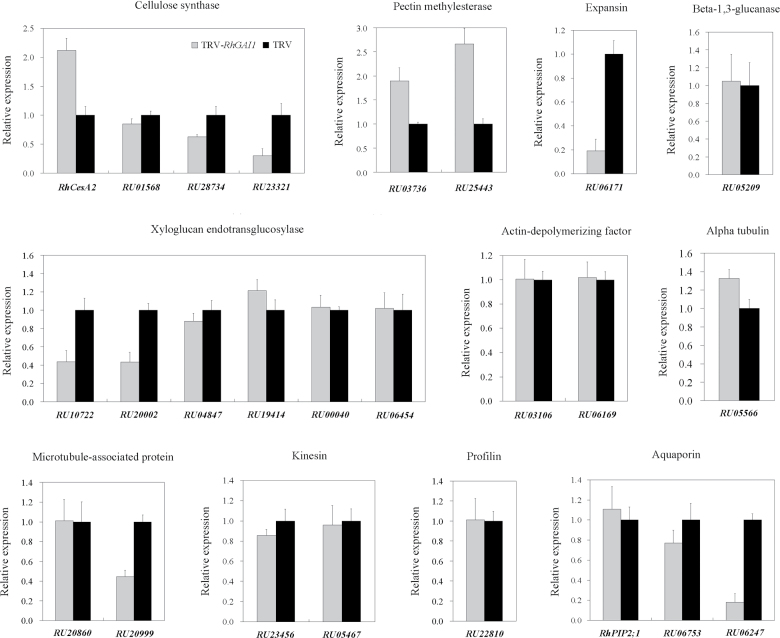
qRT-PCR analysis of 25 cell expansion-related genes in *RhGAI1*-silenced rose petals. *RhACT5* was used as internal control. The results show the mean ±SD from three biological replicates. The relative expression of the 25 genes in the TRV control was defined as 1.

In order to explore the possible mechanism of *RhGAI1*-regulated petal expansion in rose, 25 cell expansion-related genes were selected from the microarray database (Supplementary Table S4 at *JXB* online), and their expression was checked by qRT-PCR in *RhGAI1*-silenced rose petals. Among them, three genes were up-regulated, six genes were down-regulated, and the remaining 16 genes were unchanged (Fig. 6). The three up-regulated genes were *RhCesA2*, *RU03736*, and *RU25443*. The six down-regulated genes were *RU23321*, *RU06171*, *RU10722*, *RU20002*, *RU20999*, and *RU06247*.

**Fig. 7.**
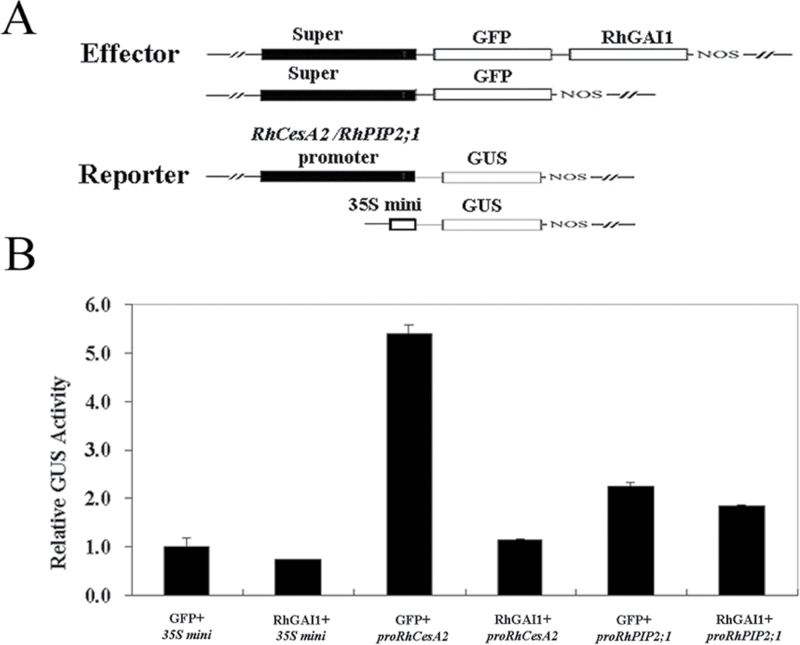
Regulation of *proRhCesA2* activity by RhGAI1 in *Arabidopsis* mesophyll protoplasts. (A) Diagrams of effector and reporter constructs. (B) Regulation of *RhCesA2* promoter activity by RhGAI1. The *35S mini* promoter was used as the control, and its relative GUS activity was defined as 1.

To test whether the genes whose expression was changed in *RhGAI1*-silenced rose petals were downstream genes of *RhGAI1*, a transactivation analysis was performed in *Arabidopsis* mesophyll protoplasts. Since the expression of *RhCesA2* was apparently enhanced in *RhGAI1*-silenced petals, while the expression of *RhPIP2;1* was not clearly changed compared with the TRV control, the promoters of *RhCesA2* and *RhPIP2;1* were used to construct the reporters (Fig. 7A). The promoter activity of *RhCesA2* was strongly repressed by RhGAI1, while the promoter activity of *RhPIP2;1* was barely changed by RhGAI1 (Fig. 7B).

### *RhCesA2* is a direct target of RhGAI1

It is reported that a GRAS transcription factor NSP1 can bind to the AATTT element in the promoter of *OND11* by EMSA during nodulation signalling in *Medicago truncatula* (Hirsch *et al.*, 2009). Since DELLA proteins are a subfamily of GRAS transcription factors, and the binding region in the GRAS domain is conserved among GRAS transcription factors, AATTT may also be the binding site of DELLA proteins.

Through sequence analysis of the *RhCesA2* promoter, six putative AATTT elements were found to be located in the promoter of *RhCesA2* (Supplementary Fig. S4A at *JXB* online). After truncation, two fragments, *proRhCesA2* D1 and *proRhCesA2* D2, were generated. The results of a transactivation assay in protoplasts showed that the activities of *proRhCesA2* D1 and *proRhCesA2* D2 dropped to 51% and 46%, respectively, when compared with *proRhCesA2*. In addition, these fragments were also strongly repressed by RhGAI1 (Supplementary Fig. S4B). These results suggested that there were important binding sites in *proRhCesA2* D2. Therefore, a second truncation was carried out to determine the positive binding site, and *proRhCesA2* D3 and *proRhCesA2* D4 were obtained (Supplementary Fig. S4A). The promoter activities of these fragments were gradually reduced, and still notably repressed by RhGAI1 (Supplementary Fig. S4C). These results indicated that *RhCesA2* might be a downstream gene of *RhGAI1*, and the AATTT element in *proRhCesA2* D4 might partly account for the repression of the promoter activity of *RhCesA2* by RhGAI1.

**Fig. 8.**
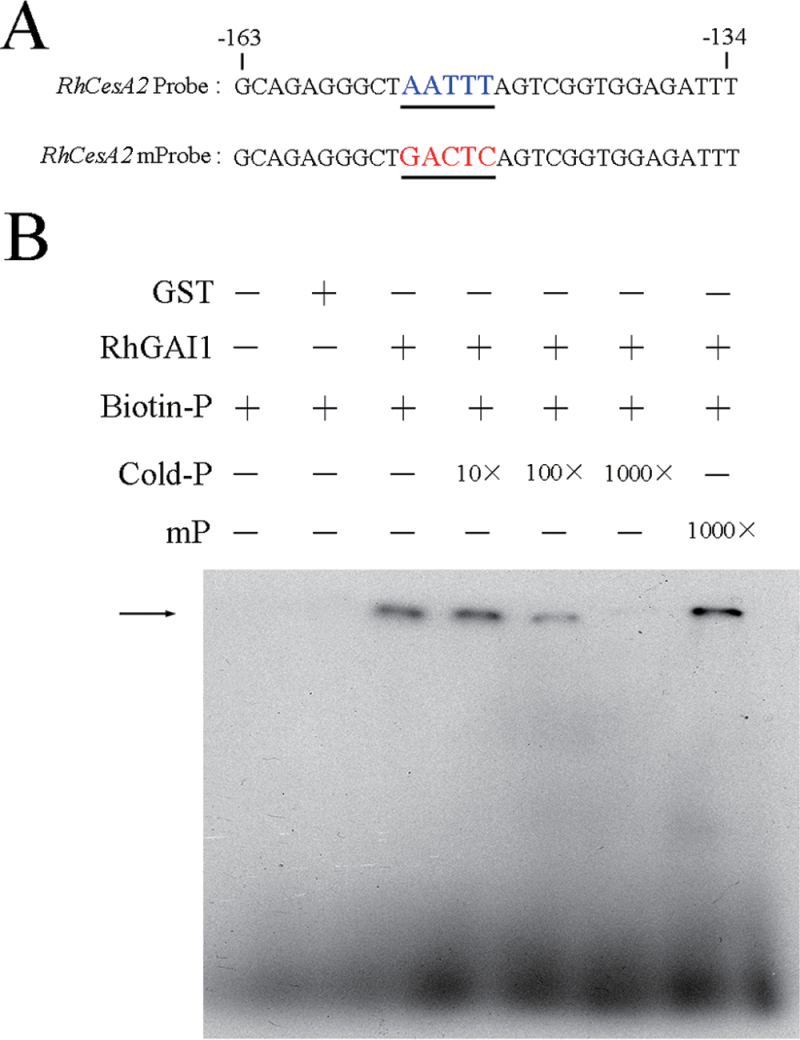
Binding test of RhGAI1 and the probe of *proRhCesA2 in vitro*. (A) The *RhCesA2* probe was labelled with biotin. (B) EMSA analysis showing the binding of RhGAI1 to the *RhCesA2* promoter. The black arrow indicates the binding of RhGAI1 and the biotin-labelled probe.

To verify whether RhGAI1 can bind directly to the promoter of *RhCesA2*, an EMSA was carried out. The biotin-labelled probe was designed according to the AATTT element in *proRhCesA2* D4 (Fig. 8A). The EMSA result showed that RhGAI1 could bind to the biotin-labelled *proRhCesA2* probe. Also, this binding diminished gradually with an increasing concentration of unlabelled probe, whereas it could not be competed by the mutated probe (Fig. 8B), indicating that *RhCesA2* is a direct target of RhGAI1.

The promoters of *RU25443* and *RU10722* were also cloned, and it was found that there were eight putative AATTT elements in the 1255bp promoter region of *RU25443* and two putative AATTT elements in the 1347bp promoter region of *RU10722*. The promoter activity of *RU25443* was strongly repressed by RhGAI1, while the promoter activity of *RU10722* was induced by RhGAI1 (Supplementary Fig. S5 at *JXB* online), which was consistent with the expression level of *RU25443* and *RU10722* in *RhGAI1*-silenced petals. They might also be downstream genes of *RhGAI1*. It is speculated that RhGAI1 may function in cell expansion through directly targeting multiple downstream genes related to cell expansion.

## Discussion

### *RhGAI1* is regulated by ethylene at the transcriptional level in rose petals

It is well known that ethylene regulates plant organ growth, such as elongation of root and hypocotyls in *Arabidopsis* and internode elongation in rice (Hall and Bleecker, 2003; Swarup *et al.*, 2007; Qi *et al.*, 2011). In addition, DELLA proteins are involved in regulation of organ growth, including cotyledon expansion and hypocotyl elongation in *Arabidopsis* (de Lucas *et al.*, 2008; Josse *et al.*, 2011). In terms of involvement of DELLA proteins in ethylene-regulated organ growth, it is reported that ethylene inhibits root elongation by enhancing the stability of DELLA proteins (Achard *et al.*, 2003). In the present work, a *DELLA* gene, *RhGAI1*, was found to be induced by ethylene, and it was a direct downstream gene of *RhEIN3-3*. This work uncovers the transcriptional regulation of *RhGAI1* by ethylene, which has not been reported before, and provides a better understanding of how ethylene regulates petal expansion in roses.

Regarding regulation of DELLA proteins, most work has focused on the post-transcriptional regulation of DELLA proteins, such as ubiquitination, phosphorylation, and *O*-GlcN acylation (Hauvermale *et al.*, 2012).

At the transcription level, it is reported that PIL5, a basic helix–loop–helix (bHLH) transcription factor, can bind directly to the promoter of *DELLA* genes, and therefore regulates GA responsiveness (Oh *et al.*, 2007).

Based on the post-transcriptional regulation of DELLA proteins by ethylene reported before and the transcriptional regulation in this work, it is speculated that, on one hand, ethylene induces the expression of *RhGAI1*; on the other hand, ethylene enhances the stability of RhGAI1, and consequently the expansion of petals is repressed.

### *RhGAI1* can regulate ethylene-responsive genes that are related to cell expansion

Since DELLA proteins play important roles in cell expansion, researchers have spent much effort in identifying its downstream genes (Cao *et al.*, 2006; Hou *et al.*, 2008). It is widely accepted that DELLA proteins do not have DNA binding capacity, and they regulate the downstream genes by interacting with other transcription factors such as PIF3 (Feng *et al.*, 2008), PIF4 (de Lucas *et al.*, 2008), JAZs (Hou *et al.*, 2010), MYC2 (Hong *et al.*, 2012), BZR1 (Gallego-Bartolomé *et al.*, 2012), EIN3 (An *et al.*, 2012), and SPL3 (Yu *et al.*, 2012).

With regard to the regulation of downstream genes by DELLA proteins, except for indirect regulation through interaction of DELLA with other transcription factors, there also exists direct transcriptional regulation (Gallego-Bartolomé *et al.*, 2011). Using ChIP-qPCR, several direct targets of DELLA proteins were found, such as *SCL3*, *MYB*, and *GID1b* (Zentella *et al.*, 2007; Zhang *et al.*, 2011).

In this work, it was found that the expression of nine cell expansion-related genes was clearly changed in *RhGAI1*-silenced rose petals when compared with the TRV control. Furthermore, *RhCesA2* was a direct downstream gene of RhGAI1. These genes are widely involved in loosening and synthesis of the cell wall, changes of turgor pressure, and remodelling of the cytoskeleton, which suggests that *RhGAI1* plays a comprehensive role in cell expansion.

In terms of synthesis of new cell wall materials, it is widely reported that *CESA* genes are involved in this process (Mutwil *et al.*, 2008). The null allele of *CESA6*, *prc1-8*, showed increases in width in both epidermal and cortical cells (Fagard *et al.*, 2000). What is more, in the *cesa2 cesa6 cesa9* triple mutant, irregular wall thickness is observed, which results in shrinkage of pollen grains (Persson *et al.*, 2007). These reports indicate that *CESA* genes play an important role in synthesis of the cell wall and cell expansion, and *RhCesA2* may be an important downstream gene in *RhGAI1*-regulated cell expansion of rose petals.

Nevertheless, the current data are insufficient to provide a complete view of the role of *RhGAI1* during cell expansion. Through a transactivation assay, the promoter activity of *RU25443* (*PE*) and *RU10722* (*XTH*) was regulated by RhGAI1, which suggested that they might also be downstream genes of *RhGAI1*. Whether there are any other downstream genes of *RhGAI1* and what their regulatory mechanisms are need further investigation.

### Accession numbers

Sequence data from this article can be found in the EMBL/GenBank data libraries under accession numbers: RhGAI1 (AGK07287), RhEIN3-3 (AGK07288), MdGAI1 (ADW85805), MhGAI1 (ABL61270), PtGAI/RGA1 (XP_002314799), GbGAI (ABG26370), RcGAI (XP_002527794), GhGAI/RGA (ACR58455), VvGAI1 (AEK06229), BoGAI (BAG16374), BnRGA (ADD71137), BrGAI (BAG16380), SsGAI (ACM47244), GmGAI 1 (NP_001240948), LsDELLA 1 (BAG71200), SlGAI (NP_001234365), RlDELLA (AFC88481), PvGAI2 (BAF62637), AmGRAS (ADA84480), PsDELLA (ABI30654), DmGAI (AAM15884), AaGAI-like 1 (ABL97842), LiGAI-like 1 (ABL97941), WgGAI (AAM15886), DcGAI (AAM15882), ApGAI-like 1 (ABL97840), DaGAI (AAM15880), VrGAI-like 1 (ABL97934), MsGAI (AAM15891), DrGAI (AAM15885), VaGAI-like 1 (ABL97928), CsGAI-like 1 (ABL97886), YtGAI-like 1 (ABL97940), VpGAI-like 1 (ABL97932), VtGAI-like 1 (ABL97938), VfGAI-like 1 (ABL97930), CmGAI (AAM15892), AkGAI (AAM15901), AtRGA (NP_178266), AtGAI (NP_172945), AtRGL1 (NP_176809), AtRGL2 (NP_186995), AtRGL3 (NP_197251), RcEIN3 (XP_002530192), AdEIL2 (ACJ70675), VrEIL2 (AAL76271), NtEIL2 (AAP03998), NtEIL1 (AAP03997), VvEIL (XP_002275284), LeEIL (ACP56697), MtEIL1 (ACX54782), NtEIL4 (AAP04000), SlEIL1 (NP_001234541), SlEIL2 (NP_001234721), AtEIN3 (NP_188713), PpEIL2 (ABK35086), CsEIN3 (AFK80347), DcEIL (BAI44821), AtEIL1 (AAC49746), AtEIL2 (NP_197611), AtEIL3 (NP_177514), SlEIL3 (NP_001234546), ZmEIL1 (NP_001152035), PtEIN3a (XP_002312841), PtEIN3b (XP_002328098), MdEIN3 (ADE41155), OsEIL (BAB78462), RhETR1 (AY953869), RhETR3 (AF154119), RhETR5 (AF441283), RhEIN3-1 (AF443783), RhEIN3-2 (AY919867), and RhCseA1 (JQ001775).

## Supplementary data

Supplementary data are available at *JXB* online.

Figure S1. Alignment of deduced amino acid sequence of RhGAI1 with nine DELLA proteins of other plants.

Figure S2. Phylogenetic tree of RhGAI1 and other DELLA proteins.

Figure S3. Phylogenetic tree of RhEIN3-3 and other EIN3/EILs proteins.

Figure S4. Transactivation of truncated *proRhCesA2* by RhGAI1.

Figure S5. Regulation of *proRU25443* and *proRU10722* by RhGAI1.

Table S1. Oligonucleotide primer sequences.

Table S2. Expression of unisequences of *DELLA* genes in rose petals by microarray analysis.

Table S3. Expression of unisequences of *EIN3* genes in rose petals by microarray analysis.

Table S4. Expression of unisequences of cell expansion-related genes in rose petals by microarray analysis.

Supplementary Data

## Abbreviations:

AbsE: axial subepidermis
CESA: cellulose synthase
EBS: EIN3 binding site
GA: gibberellic acid
GAI: GA INSENSITIVE
GID1: GA-INSENSITIVE DWARF1
MAP: microtubule-associated protein
PE: pectin esterase
RACE: rapid amplification of cDNA ends
RGA: REPRESSOR OF ga1-3
RGL: RGA-LIKE protein
TIP: tonoplast intrinsic protein
TRV: *Tobacco rattle virus*
UTR: untranslated region
VIGS: virus-induced gene silencing
XTH: xyloglucan endotransglycosylase.
