# Supplementary material for: A DELLA gene, RhGAI1, is a direct target of EIN3 and mediates ethylene-regulated rose petal cell expansion via repressing the expression of RhCesA2
Source: J Exp Bot. 2013 Sep 7;64(16):5075–84. doi: 10.1093/jxb/ert296 (PMC3830487; doi:10.1093/jxb/ert296)
Supplement: Supplementary Data [file supp_64_16_5075__index.html]

A DELLA gene, RhGAI1, is a direct target of EIN3 and mediates ethylene-regulated rose petal cell expansion via repressing the expression of RhCesA2 — A DELLA gene, RhGAI1, is a direct target of EIN3 and mediates ethylene-regulated rose petal cell expansion via repressing the expression of RhCesA2 — Supplementary Data 

# A *DELLA* gene, *RhGAI1*, is a direct target of EIN3 and mediates ethylene-regulated rose petal cell expansion via repressing the expression of *RhCesA2*

## Supplementary Data

Data files

**Files in this Data Supplement:**

- Supplementary Data - Supplementary Data
