# Supplementary material for: A DELLA gene, RhGAI1, is a direct target of EIN3 and mediates ethylene-regulated rose petal cell expansion via repressing the expression of RhCesA2
Source: J Exp Bot. 2013 Sep 7;64(16):5075–84. doi: 10.1093/jxb/ert296 (PMC3830487; doi:10.1093/jxb/ert296)
Supplement: Supplementary Data [file supp_ert296_jexbot103051_file001.pdf]

A *DELLA* gene, *RhGAI1*, is a direct target of EIN3 and mediates ethylene-regulated rose petal cell expansion via repressing the expression of *RhCesA2*

Jing Luo, Nan Ma, Haixia Pei, Jiwei Chen, Jing Li, Junping Gao*

Supplementary Figures

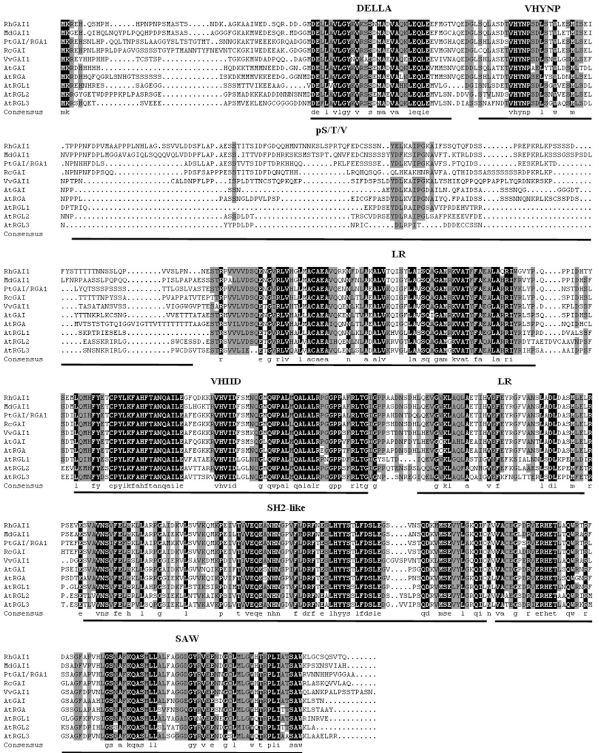

Supplementary Fig. S1. Alignment of deduced amino acid sequence of RhGAI1 with nine DELLA proteins of other plants. The lines indicate the conserved domain of RhGAI1, including DELLA, VHYNP, pS/T/V, LR, VHIID, LR, SH2-Like, and SAW. The nine DELLA proteins from other plants includes MdGAI1, PtGAI/RGA1, RcGAI, VvGAI1, AtRGA, AtGAI, AtRGL1, AtRGL2, AtRGL3.

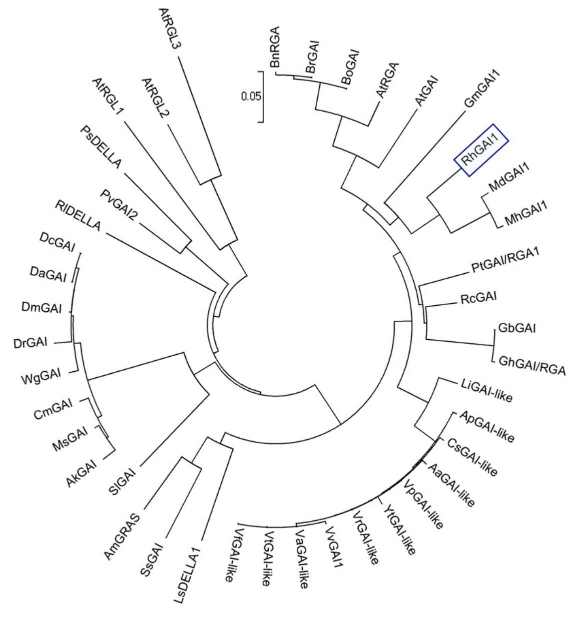

Supplementary Fig. S2. Phylogenetic tree of RhGAI1 and other DELLA proteins. The RhGAI1 protein is aligned with 41 DELLA proteins of other plants, including MdGAI1, MhGAI1, PtGAI/RGA1, GbGAI, RcGAI, GhGAI/RGA, VvGAI1, BoGAI, BnRGA, BrGAI, SsGAI, GmGAI1, LsDELLA1, SlGAI, RlDELLA, PvGAI2, AmGRAS, PsDELLA, DmGAI, AaGAI-like1, LiGAI-like1, WgGAI, DcGAI, ApGAI-like1, DaGAI, VrGAI-like1, MsGAI, DrGAI, VaGAI-like1, CsGAI-like1, YtGAI-like1, VpGAI-like1, VtGAI-like1, VfGAI-like1, CmGAI, AkGAI, AtRGA, AtGAI, AtRGL1, AtRGL2, AtRGL3.

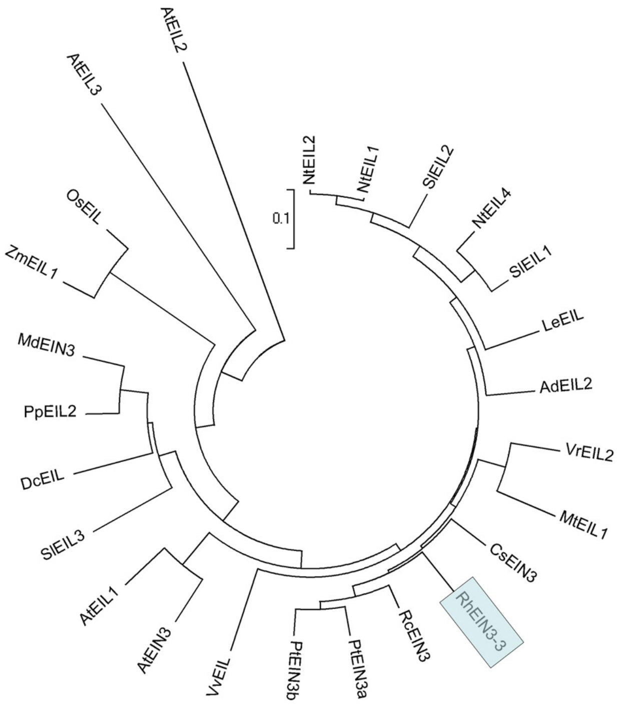

Supplementary Fig. S3. Phylogenetic tree of RhEIN3-3 and other EIN3/EILs proteins. The RhEIN3-3 protein is aligned with 24 EIN3/EILs proteins from other plants, including RcEIN3, AdEIL2, VrEIL2, NtEIL2, NtEIL1, VvEIL, LeEIL, MtEIL1, NtEIL4, SlEIL1, SlEIL2, AtEIN3, PpEIL2, CsEIN3, DcEIL, AtEIL1, AtEIL2, AtEIL3, SlEIL3, ZmEIL1, PtEIN3a, PtEIN3b, MdEIN3, OsEIL.

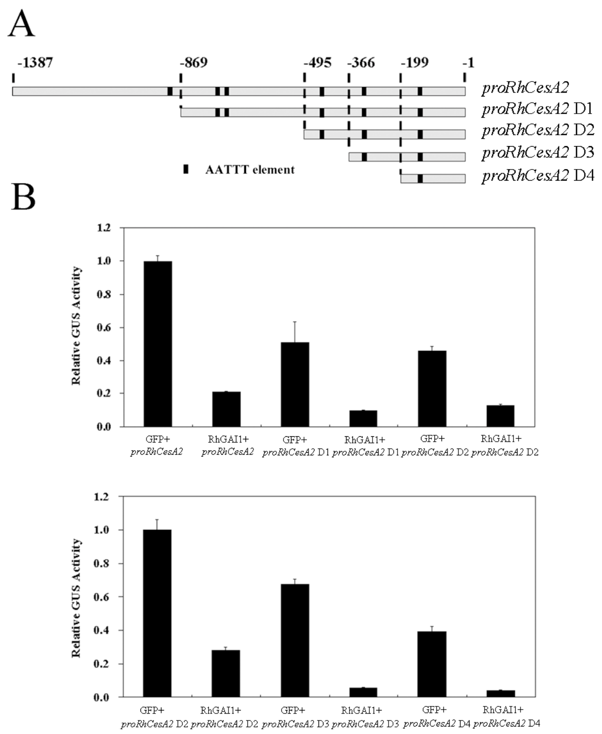

Supplementary Fig. S4. Transactivation of truncated *proRhCesA2* by RhGAI1. **(A)** Diagrams of truncation of *proRhCesA2*. The black boxes indicate the AATTT elements. **(B)** and **(C)** Regulation of truncated promoter activity of *RhCesA2* by RhGAI1. Different combinations of effector and reporter were co-transferred into protoplasts, and the relative GUS activity indicates the promoter activity.

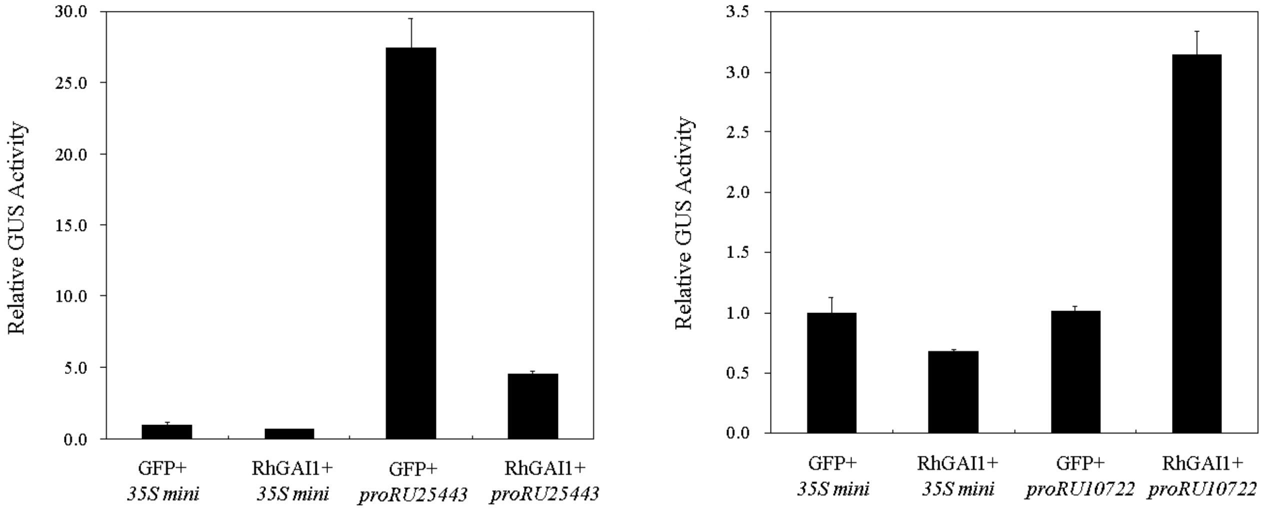

Supplementary Fig. S5. Regulation of *proRU25443* and *proRU10722* by RhGAI1. Arabidopsis mesophyll protoplasts were co-transfected with different combinations of effector and reporter, and the relative GUS activity indicated the promoter activity. The *35S mini* promoter was used as the control, and its relative GUS activity in *35S mini* promoter and GFP co-transfected protoplasts was defined as 1.

Supplementary Tables

Supplementary Table S1. Oligonucleotide primer sequences.

Primers	Sequences
**For Vector Construction**	
RhETRs VIGS Up	5’-CGTCTAGACAGAAGCACTCTTGATCGACAT-3’
RhETRs VIGS Low	5’-TACCCGGGTTTGACAGAGGCAACAGGC-3’
RhGAI1 VIGS Up	5’-GTA TCTAGA CACTGAGTCTGGTCAACG-3’
RhGAI1 VIGS Low	5’-TAT GGTACC ATTATCACCACCAACCTAC-3’
RhGAI1 OE Up	5’-GCGCTCGAGATGAAAAGAGAACATCAAAGCC-3’
RhGAI1 OE Low	5’-TAT ACTAGT TCA CTG CGT CAC CGA TTG-3’
Rhgai1 F1	5’-GCGCTCGAGATGAAAAGAGAACATCAAAGCC-3’
Rhgai1 R1	5'-GCTTCTGGGCCACATCCATGCCGTCGTCGCG-3'
Rhgai1 F2	5'-GACGACGGCATGGATGTGGCCCAGAAGCTC-3'
Rhgai1 R2	5’-TAT ACTAGT TCA CTG CGT CAC CGA TTG-3’
proRhGAI1 Up	5'-CTGAAGCTTCCGACCCAGTAATTGTGTGG-3'
proRhGAI1 Low	5'-GCCGGATCCGATTTGCTTTGGTAATTAGCTCG-3'
proRhPIP2;1 Up	5'-CTGAAGCTTGTTGTTGCAATCCCGGTAC-3'
proRhPIP2;1 Low	5'-GCGAAGCTTCAACACTGGAATAGTTGGC-3'
proRhCesA1 Up	5'-TCTAAGCTTCACTGTCCAGAAGGGACG-3'
proRhCesA1 D1 Up	5'-GCGAAGCTTCCTTCGTGGGTAATACTCACTAG-3'
proRhCesA1 D2 Up	5'-ATTAAGCTTGACAGTGCTGATGGTCCCTAG-3'
proRhCesA1 D3 Up	5'-TATAAGCTTTGTGTCGGCGGAGATATTG-3'
proRhCesA1 D4 Up	5'-GCGAAGCTTAAGAGAGGAAGAAAGGAATGATAAG-3'
proRhCesA1 Low	5'-GATAAGCTTGCTTTAAGCAACCAAAGCT-3'
**For qRT PCR**	
RhGAI1 RT Up	5'-ACCACCCTAATCCAAACCCTTC-3'
RhGAI1 RT Low	5'-AGCCCGTCTTCCTGAACTGTC-3'
RhETR1 RT Up	5'-ACCACTCGTGGCAAGATATGTG-3'
RhETR1 RT Low	5'-CTCGCCACTTTCTAGTGCTATCTG-3'
RhETR2 RT Up	5'-TTATCGCGTTTATCGTGCAATG-3'
RhETR2 RT Low	5'-TTGAGAAGCAAAGGAATGAGAGTG-3'
RhETR3 RT Up	5'-TGTATTGTGTGGAGCGACACATC-3'
RhETR3 RT Low	5'-GAATCAAACCCATTTCTCTGTCG-3'
RhActin5 RT Up	5'-AAGGTGGTTGCCCCACCAGA-3'
RhActin5 RT Low	5'-ACCAGAGAACAAGAATGCAAGCAGC-3'

PIP2;1 RT Up	5'-TAAGGTCTCTCTGATCCGGGC-3'
PIP2;1 RT Low	5'-GTGGCTGAGAAGACGGTGTAGAC-3'
RU03106 RT Up	5'-CATTGGCAATGCAATTTGGAG-3'
RU03106 RT Low	5'-TTGGCCATAAGTTTGTATTTCACAC-3'
RU03736 RT Up	5'-ACCCTCTACCCCGAGCTCTG-3'
RU03736 RT Low	5'-CGCTTCGTGAGGCTCTTCAC-3'
RU04718 RT Up	5'-GCAACCATAGCCTCAATCACG-3'
RU04718 RT Low	5'-GTTGTACTTGTGCCCAACAGTCC-3'
RU04847 RT Up	5'-GCTTGGCACCTTTCAGATTCTC-3'
RU04847 RT Low	5'-AAGATGCCATGCAAGAGACCC-3'
RU05566 RT Up	5'-ATATCATCCGTGTCTAGCCAGTTAG-3'
RU05566 RT Low	5'-ATGAACACTCAACATTAAAGCTTCG-3'
RU20999 RT Up	5'-GAGATCTCGGTCTCGAGTAGTTCC-3'
RU20999 RT Low	5'-GGAGGTCAAATAGGTTGGCTTG-3'
RU01568 RT Up	5'-CCCAACTAAAGCTCGAGATTTAGAG-3'
RU01568 RT Low	5'-AACAAATCTATTTTTCCCCTCGAC-3'
RU02147 RT Up	5'-AAGCTGGCTCAACTCTTTACCTG-3'
RU02147 RT Low	5'-CATTACGATCCCAGAAACTGTGTG-3'
RU06171 RT Up	5'-GGTAGTTCGGTCAAACATTTTCAG-3'
RU06171 RT Low	5'-CACCTCAGCAAAACACGCC-3'
RU06247 RT Up	5'-CCCTCAGAACCAGTAGTGAAG-3'
RU06247 RT Low	5'-ATGAGAGTGGAGATGAACTCGG-3'
RU06753 RT Up	5'-AACATGTGCTCTTCTGTGGGTATTC-3'
RU06753 RT Low	5'-GTCAGGGAGAGTTTCCTTGCC-3'
RU10722 RT Up	5'-GATGGGGATTTCTGGTAACGTG-3'
RU10722 RT Low	5'-TGACCTCCCTTGAATATCTTAGGAC-3'
RU18017 RT Up	5'-GCTTCACTGTCACAGCCAAGTC-3'
RU18017 RT Low	5'-CACCCAGAAGGCAAAGAAGAG-3'
RU20860 RT Up	5'-AGTTGTTGTGAGTAGTGCTACACCG-3'
RU20860 RT Low	5'-GCATTCCATTTTGTGAGCTACC-3'
RU23456 RT Up	5'-CCAGAGTTTCCTCCATGTCACC-3'
RU23456 RT Low	5'-AATTGGTTCCAACGTCCACG-3'
RU28734 RT Up	5'-GAAGTAGCATCTCCTTTGCCTGTC-3'
RU28734 RT Low	5'-AGGAAGGTCACACCTTGGAAGAG-3'

Supplementary Table S2. Expression of unisequences of *DELLA* genes in rose petals by microarray analysis.

Probe_Name	unigene ID	GenBank top hit			ethylene vs control									
					time 1		time 2		time 3		time 4		time 5	
		Accession	Description	evalue	Ratio	FDR	Ratio	FDR	Ratio	FDR	Ratio	FDR	Ratio	FDR
yueji_05899	RU57863	ABL61270	GAI1 [Malus hupehensis]	4.00E-27	2.371	0.000	2.350	0.000	1.076	0.811	1.623	0.033	1.518	0.034
yueji_12651	RU01371	AAY56752	DELLA protein [Malus x dome	4.00E-101	2.267	0.000	2.183	0.000	1.134	0.638	1.442	0.063	1.461	0.037
yueji_14949	RU24255	ABV24890	GAI-like protein [Rosa gallica	5.00E-17	2.124	0.000	2.045	0.000	1.055	0.831	1.131	0.457	1.413	0.015
yueji_04318	RU21140	ABV24890	GAI-like protein [Rosa gallica	1.00E-57	2.102	0.000	1.739	0.005	1.032	0.937	1.274	0.299	1.410	0.060
yueji_01330	RU03348	NP_0012343	DELLA protein GAI [Solanum	1.00E-23	2.086	0.000	1.915	0.000	1.188	0.392	1.411	0.039	1.338	0.068
yueji_03938	RU08333	AAY56753	DELLA protein [Malus x dome	1.00E-28	1.017	0.912	0.531	0.000	0.436	0.000	0.553	0.001	0.455	0.000
yueji_00978	RU13323	ACV52013	GAI/RGA-like 3-a [Gossypium	2.00E-52	0.705	0.072	0.371	0.000	0.371	0.000	0.487	0.001	0.280	0.000
yueji_14371	RU08548	XP_0036074	GAI-like protein [Medicago tru	5.00E-36	0.450	0.000	0.854	0.416	0.942	0.749	0.910	0.716	1.273	0.145

Supplementary Table S3. Expression of unisequences of *EIN3* genes in rose petals by microarray analysis.

Probe_Name	unigene ID	GenBank top hit			ethylene vs control									
					time 1		time 2		time 3		time 4		time 5	
		Accession	Description	evalue	Ratio	FDR	Ratio	FDR	Ratio	FDR	Ratio	FDR	Ratio	FDR
yueji_01680	RU05931	BAB64344	EIN3-like protein [Cucumis me	1.00E-59	1.109	0.311	1.277	0.011	1.116	0.292	0.977	0.844	1.07	0.556
yueji_11090	RU03799	BAB64344	EIN3-like protein [Cucumis me	6.00E-121	0.951	0.788	1.224	0.175	1.346	0.057	0.943	0.708	1.182	0.267
yueji_02698	RU17627	CAC09582	EIN3/EIL-like [Fagus sylvatica	2.00E-59	1.316	0.035	1.171	0.186	1.245	0.090	1.223	0.089	1.381	0.005
yueji_03391	RU16486	AAL14267	EIN3-like [Rosa hybrid cultivar	1.00E-65	0.772	0.007	0.879	0.164	1.158	0.148	0.926	0.412	0.957	0.613
yueji_03980	RU53589	ABK35085	EIL1 [Prunus persica]	4.00E-32	1.934	0.000	1.316	0.030	0.938	0.744	1.152	0.266	1.071	0.621
yueji_08686	RU23484	ABK35085	EIL1 [Prunus persica]	6.00E-101	0.964	0.749	1.231	0.062	1.256	0.070	1.194	0.113	1.226	0.055
yueji_01396	RU22863	ABK35086	EIL2 [Prunus persica]	1.00E-102	1.153	0.113	0.838	0.038	0.854	0.111	0.921	0.381	0.895	0.180

Supplementary Table S4. Expression of unisequences of cell expansion-related genes in rose petals by microarray analysis.

Probe_Name	unigene ID	GenBank top hit			ethylene vs control									
					time 1		time 2		time 3		time 4		time 5	
		Accession	Description	evalue	Ratio	FDR	Ratio	FDR	Ratio	FDR	Ratio	FDR	Ratio	FDR
yueji_08365	RU03106	ACB32233	actin-depolymerizing factor	3.00E-66	0.905	0.399	0.831	0.095	1.058	0.687	1.141	0.224	2.107	0.000
yueji_13703	RU03736	AAR03544	pectin methylesterase isof	1.00E-121	0.876	0.547	0.563	0.012	0.969	0.867	0.601	0.029	0.807	0.343
yueji_05838	RU04718	AAP40636	cellulose synthase 6 [Popu	2.00E-71	0.915	0.507	0.618	0.000	0.546	0.000	0.650	0.001	0.502	0.000
yueji_06177	RU04847	ABM91068	xyloglucan endotransglyco	1.00E-72	1.322	0.179	0.534	0.002	0.420	0.000	0.470	0.001	0.391	0.000
yueji_01422	RU05566	AAO63781	alpha-tubulin 1 [Populus tr	9.00E-260	0.962	0.906	0.788	0.245	0.587	0.015	0.810	0.357	0.651	0.016
yueji_01709	RU20999	XP_002520	Microtubule-associated pro	7.00E-64	1.470	0.080	1.563	0.047	1.254	0.370	1.511	0.048	2.308	0.000
yueji_11804	RU01568	ABY25274	cellulose synthase [Eucaly	2.00E-154	0.992	0.984	0.728	0.008	0.584	0.000	0.875	0.327	0.631	0.000
yueji_06248	RU02147	CAC94006	endo-beta-1,4-glucanase [	0	0.752	0.186	0.683	0.058	0.609	0.025	0.631	0.028	0.509	0.001
yueji_10298	RU06171	AAF21101	expansin [Fragaria x anana	1.00E-110	0.782	0.252	0.328	0.000	0.730	0.190	0.356	0.000	0.761	0.114
yueji_02965	RU06247	BAB12722	gamma tonoplast intrinsic	4.00E-126	1.098	0.810	0.430	0.006	0.301	0.001	0.325	0.003	0.308	0.000
yueji_13065	RU06753	ABD63904	aquaporin 1 [Gossypium h	1.00E-92	0.943	0.847	0.551	0.019	0.362	0.001	0.309	0.000	0.225	0.000
yueji_04478	RU10722	P35694	Brassinosteroid-regulated	3.00E-57	0.690	0.299	0.442	0.081	0.452	0.276	0.275	0.008	0.214	0.001
yueji_01647	RU18017	ACC59196	cellulose synthase [Betula	8.00E-36	0.869	0.206	0.709	0.002	0.708	0.005	0.839	0.130	0.646	0.000
yueji_05109	RU20860	XP_002511	Microtubule-associated pro	1.00E-41	2.953	0.001	4.400	0.000	2.446	0.004	4.645	0.000	5.376	0.000
yueji_02463	RU23456	BAB11329	kinesin-like protein [Arabid	6.00E-36	0.800	0.146	0.510	0.000	0.441	0.000	0.628	0.007	0.432	0.000
yueji_11012	RU28734	NP_180039	Cellulose synthase-like[Ara	2.00E-25	0.915	0.798	0.352	0.001	0.293	0.001	0.359	0.003	0.316	0.000
yueji_09140	RU20002	ABE73119	endo-transglycosylase [Dia	3.00E-09	0.592	0.345	0.226	0.042	5.674	0.008	1.722	0.295	4.869	0.007
yueji_06490	RU19414	ABM91064	xyloglucan endotransglyco	5.00E-25	0.897	0.708	0.446	0.013	0.563	0.244	0.304	0.001	0.622	0.126
yueji_00469	RU00040	AAZ08349	xyloglucan endotransglyco	1.00E-15	0.964	0.790	0.440	0.020	0.617	0.270	3.042	0.006	0.529	0.056
yueji_10791	RU05467	NP_195616	kinesin-related protein (MK	2.00E-38	0.971	0.820	0.863	0.171	0.704	0.004	0.782	0.030	0.627	0.000
yueji_06591	RU06454	ABB86296	xyloglucan endotransgluco	1.00E-178	1.331	0.436	1.809	0.067	1.805	0.052	1.421	0.293	2.819	0.001
yueji_07830	RU05209	AAY96764	1,3-beta-D-glucanase [Pha	2.00E-105	0.741	0.687	1.597	0.488	9.253	0.000	4.026	0.005	2.774	0.024
yueji_06597	RU23321	ACJ38667	cellulose synthase [Betula	3.00E-69	0.806	0.174	0.518	0.000	0.519	0.000	0.627	0.005	0.513	0.000
